# A computational approach to discovering the functions of bacterial phytochromes by analysis of homolog distributions

**DOI:** 10.1186/1471-2105-7-141

**Published:** 2006-03-16

**Authors:** Tilman Lamparter

**Affiliations:** 1 Freie Universität Berlin, Pflanzenphysiologie, Königin-Luise Str. 12-16, D-14195 Berlin, Germany

## Abstract

**Background:**

Phytochromes are photoreceptors, discovered in plants, that control a wide variety of developmental processes. They have also been found in bacteria and fungi, but for many species their biological role remains obscure. This work concentrates on the phytochrome system of *Agrobacterium tumefaciens*, a non-photosynthetic soil bacterium with two phytochromes. To identify proteins that might share common functions with phytochromes, a co-distribution analysis was performed on the basis of protein sequences from 138 bacteria.

**Results:**

A database of protein sequences from 138 bacteria was generated. Each sequence was BLASTed against the entire database. The homolog distribution of each query protein was then compared with the homolog distribution of every other protein (target protein) of the same species, and the target proteins were sorted according to their probability of co-distribution under random conditions. As query proteins, phytochromes from *Agrobacterium tumefaciens*, *Pseudomonas aeruginosa*, *Deinococcus radiodurans *and *Synechocystis *PCC 6803 were chosen along with several phytochrome-related proteins from *A. tumefaciens*. The *Synechocystis *photosynthesis protein D1 was selected as a control. In the D1 analyses, the ratio between photosynthesis-related proteins and those not related to photosynthesis among the top 150 in the co-distribution tables was > 3:1, showing that the method is appropriate for finding partner proteins with common functions. The co-distribution of phytochromes with other histidine kinases was remarkably high, although most co-distributed histidine kinases were not direct BLAST homologs of the query protein. This finding implies that phytochromes and other histidine kinases share common functions as parts of signalling networks. All phytochromes tested, with one exception, also revealed a remarkably high co-distribution with glutamate synthase and methionine synthase. This result implies a general role of bacterial phytochromes in ammonium assimilation and amino acid metabolism.

**Conclusion:**

It was possible to identify several proteins that might share common functions with bacterial phytochromes by the co-distribution approach. This computational approach might also be helpful in other cases.

## Background

Many photoreceptors such as rhodopsins, phytochromes, cryptochromes and phototropins have been discovered in eukaryotic organisms by a combination of biochemical and physiological assays [1], whereas prokaryotic versions of these proteins have often been identified during genome projects. Phytochromes, which are photoreceptors with a bilin chromophore, control a broad range of developmental processes in plants [2]. The discovery of plant phytochrome in the late 1950s was the starting point for biochemical, molecular and physiological characterisations. Since the late 1990s, phytochromes have also been found in many bacteria and fungi [3-8], in most cases after the phytochrome gene has been identified during genome sequencing [9]. Prototypical bacterial phytochromes are light-regulated histidine kinases, which trans-phosphorylate cognate response regulator proteins [4]. In cyanobacteria, a number of light effects such as phototaxis [10], control of the circadian clock [11], chromatic adaptation [12] and adaptation to blue light conditions [13], are controlled by proteins that contain domains with rather weak homology to the so-called GAF-domain of phytochrome. The biological function of prototypical phytochrome is known for only a few bacteria. The cyanobacterial phytochrome Cph1 is important for adaptation to strong light conditions [14] and is involved in regulating several genes, including *gifA*, which encodes a regulator of glutamine synthase [15]. However, the signal transduction link between Cph1 and the observed light effects is still obscure and the biological role of the prototypical phytochromes in other cyanobacteria is unknown.

The most obvious effects controlled by bacterial phytochromes have been found for *Bradyrhizobium *spp., a photosynthetic plant symbiont, and the purple bacterium *Rhodopseudomonas palustris*. In both species, the synthesis of bacteriochlorophyll and carotenoid pigments is under phytochrome control [16,17].

Many non-photosynthetic bacteria, including the gamma ray resistant *Deinococcus radiodurans*, the soil bacterium *Agrobacterium tumefaciens *and the pathogen *Pseudomonas aeruginosa *also contain phytochromes [5,6,18]. For *D. radiodurans*, it has been reported that the phytochrome BphP controls the regulation of carotenoid biosynthesis [5], but as in *Synechocystis*, the signal transduction link between input and output is unknown. The biological role of phytochromes in other non-photosynthetic bacteria is unknown.

The present work concentrates on the phytochrome system of *A. tumefaciens*. This bacterium is known for its ability to induce plant tumours by gene transformation, a mechanism that is widely used for plant transformation [19]. The genome of this bacterium was sequenced by two groups in 2001 [20,21], the sequencing data revealed two phytochrome genes. Since their discovery, both *A. tumefaciens *phytochromes have been analysed biochemically as recombinant proteins. These studies provided deeper insight into general phytochrome functions and phytochrome diversity [6,22-28]. Both phytochromes are expressed in *A. tumefaciens *cells, as revealed by UV/vis spectroscopy [29].

The computational approach presented here is aimed at identifying proteins that might share common features with bacterial phytochromes. The analysis is based on the distribution of protein homologs among bacteria according to the following concept: if proteins share common functions in one organism, they might have evolved together, with similar mutation rates, and their homologs should be abundant in similar subsets of species.

## Results

**Figure 1:**
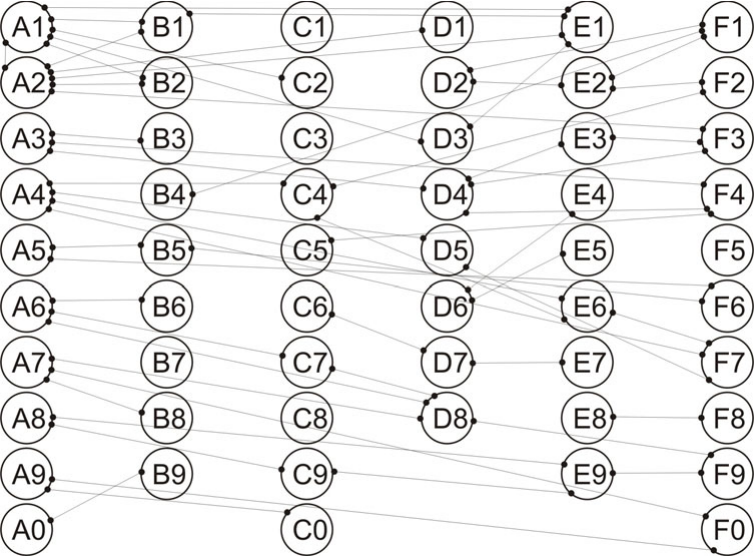
**Schematic diagram for virtual co-distribution analysis**. Each circle represents a protein which is designated with a letter and a digit. The letter stands for one of six different species. Protein homologs that arise from a global BLAST analyses are connected with lines. The virtual co-distribution analysis was performed with protein A1 as a query and all proteins of species A were used as target proteins. The species in which homologs of A-proteins are found are as follows: A1: B, C, D, E; A2: B, D, E, F; A3: B, D, F; A4: C, D, E, F; A5: B, F; A6: B, C, D; A7: B, D, F; A8: C, E; A9: C, F; A0: B.

The principle of the investigation is outlined in Figure 1. In this example, six species were used for an imaginary global homology analysis. Each species has between eight and ten proteins, which are abbreviated by a letter for the species and a digit. Protein A1 of species A is the query protein of the imaginary co-distribution analysis. This protein has homologs in four other species and one homolog, A2, in species A itself. In Table 1, which gives the result of the co-distribution analysis, the proteins of species A are sorted according to the co-distribution probability *p*, which is calculated as described in the Methods section. Proteins that have homologs in a similar subset as the query protein A1 are listed at the top. Protein A6 has the best co-distribution match, with a probability of co-distribution under random conditions of p = 0.2. A2 is a direct homolog of A1; this is indicated by "1" in column e. The header of Table 1 contains further comments.

The co-distribution analyses in this study are based on protein sequences from 138 bacteria, listed in Additional file 21. The tabulated results of two global BLAST [30] searches with E-values of 0.000001 and 10 were stored in files that were used to compare the homolog distribution of protein couples. Two different BLAST analyses were undertaken in order to determine the dependence of the results on the E-value. The number of homologs usually differs according to the chosen E-value and lies within a reasonable range for the query proteins (see e.g. Table 2). Each query protein was probed against all the other proteins of the same species to produce sorted co-distribution tables, as outlined in the example of Figure 1 and Table 1 (see Additional files 1 to 20).

**Figure 2:**
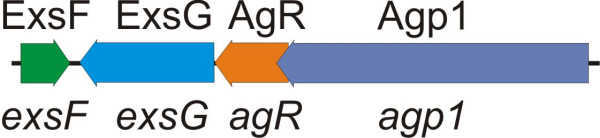
***A. tumefaciens* gene arrangement around agp1**. Gene names are listed below and names of the encoded proteins listed above the diagram. The *apg1*, *agR *and *exsG *open reading frames belong to the same operon, the *exsF *open reading frame points to the opposite direction.

The photosynthetic protein D1 from the cyanobacterium *Synechocystis *PCC 6803 was chosen as a control query protein to see whether the co-distribution approach identifies related proteins (see Additional files 17 and 18). In addition, five phytochromes from four different species were selected as query proteins: Agp1 (= BphP1) (see Additional files 1 and 2) and Agp2 (= BphP2) (see Additional files 3 and 4) from *A. tumefaciens*, BphP from *P. aeruginosa *(see Additional files 13 and 14), BphP from *D. radiodurans *(see Additional files 11 and 12) and Cph1 from *Synechocystis *(see Additional files 15 and 16). The *agp1 *gene of *A. tumefaciens *is arranged in a gene cluster as depicted in Fig. 2. A response regulator protein termed AgR and a histidine kinase termed ExsG are encoded in the same operon. It has been shown that AgR is phosphorylated by Agp1 in a light-dependent manner [23]. ExsG is homologous to the histidine kinase module of Agp2. A response regulator termed ExsF is encoded in the other DNA strand next to the *exsG *gene. In prokaryotes, such an arrangement points to possible common functions among the encoded proteins. Since the principal goal of this study was to gain information about the phytochrome system of *A. tumefaciens*, AgR (see Additional files 5 and 7), ExsF (see Additional files 7 and 8) and ExsG (see Additional files 9 and 10) were also selected as query proteins. The response regulator of *Synechocystis *phytochrome, Rcp1, which is phosphorylated by Cph1 [4], was also included (see Additional files 19 and 20).

For each query protein, the results of both global BLAST analyses were taken as sources for the co-distribution analyses. Thus, 20 co-distribution lists were generated. The target proteins in these lists were sorted as outlined in Table 1. All co-distribution lists are presented as additional files on the BMC web server.

The BLAST analysis with an E-value of 0.000001 revealed D1 homologs in 6 species. These species are identical to the 6 cyanobacteria that were selected for global analysis. In *Synechocystis*, there are 142 proteins with exactly the same distribution. Among these are 37 other proteins with photosynthesis-related functions, such as phycocyanin, allophycocyanin, ferredoxin and photosystem subunit proteins. A green background marks the corresponding field in the co-distribution table (see Additional file 17). Twenty-four of the 142 proteins have been annotated as "hypothetical" and 70 as "unknown protein". Eleven proteins that are clearly not related to photosynthesis, such as ribosomal proteins or tRNA synthetase, also have the same distribution as D1. A yellow background marks these fields in the co-distribution table.

When the D1 co-distribution analysis was based on a BLAST E-value of 10 (see Additional file 18), D1 homologs were found in 16 species. Among these are the six cyanobacteria and one other photosynthetic bacterium, *R. palustris*. The other 9 species are non-photosynthetic. Owing to the higher number of species with D1 homologs, the co-distribution results are more differentiated. For comparison with the previous survey, the top 149 proteins were inspected in more detail. Since the proteins placed between positions 141 and 149 have the same distribution, it was not possible to use exactly the same number of proteins as in the previous case. Among the top 149 proteins there are 42 photosynthesis-related proteins, 10 proteins with functions not related to photosynthesis, 17 "hypothetical proteins", 77 "unknown proteins", and 3 proteins for which the function could not clearly be assigned to photosynthesis. The number of photosynthesis-related proteins is comparable with the first analysis, but the selection is slightly different. For example, a ribulose-bisphosphate carboxylase subunit and a carbon dioxide concentrating protein subunit are among the top 149 in the second analysis, whereas the same proteins are placed at positions 184 and 378, respectively, in the first analysis.

The D1 analysis showed that proteins with related functions can be identified by the present approach, since among the top ca. 150 proteins there are three to four times more photosynthesis-related proteins than proteins with other known functions. It seems likely that among the hypothetical and unknown proteins listed at the top of the co-distribution tables, there are many other proteins with functions related to photosynthesis.

In cases where the phytochromes or phytochrome-related proteins mentioned above were chosen as query (see Additional files 1, 2, 3, 4 and 11, 12, 13, 14, 15, 16), the proteins placed within the top 100 in each co-distribution list were compared with all proteins from the same species. Text-based searches were performed to count the number of proteins belonging to particular groups of proteins such as histidine kinases, response regulators and transcription factors. These results are summarized in Table 2. It is remarkable that in all cases the frequency of histidine kinases (or "two-component sensors") among the top 100 co-distributed proteins is much higher than among all proteins of the species. For example, Agp1 has 18% to 20% co-distributed "two-component sensors" (= histidine kinases) among the top 100, whereas only 1.08% of all *A. tumefaciens *proteins belong to this group. Since bacterial phytochromes are also histidine kinases, the co-distribution with other histidine kinases might simply be based on direct homology, as between A1 and A2 in the example of Fig. 1. However, most co-distributed histidine kinases are not direct BLAST homologs, as indicated in column "e" of the co-distribution tables. The results for the co-distribution of (two-component-) response regulators, which are substrates of histidine kinases, are qualitatively comparable with those for the histidine kinases. There are, however, two exceptions: in the Agp2 and Rcp1 analyses, which were based on the low E-value BLAST search, the frequency of response regulators among the top 100 in the lists was comparable with that in the entire protein population.

In the case of the *A. tumefaciens *query proteins, the possibility was tested that proteins designated "transcriptional regulators" are enriched within the top 100 in the lists. For all five query proteins (see Additional files 1 to 10), the frequency of co-distributed transcriptional regulators increases when the BLAST analysis E-value is changed from low to high. The latter but not the former values are above average.

The five *A. tumefaciens *query proteins were also tested for co-distribution among each other. Table 3 gives the positions in the co-distribution tables for each possible combination. This table shows that AgR and Agp1 match quite well: in three out of four combinations, the target protein was among the first 100 in the co-distribution list. Similarly, there is also a rather good match between Cph1 and Rcp1 of *Synechocystis *(Table 4). The putative response regulator of *D. radiodurans *BphP (gi number: 15807719) appears at positions 5 and 433 in the BphP co-distribution tables. For the combinations ExsG/Agp2, ExsF/Agp2 and ExsF/ExsG, the target protein is placed among the first 100 in the co-distribution lists, indicating a rather high co-distribution. Agp2 and ExsG are direct BLAST homologs, as indicated in column "e" of the result tables. The similarity between these two proteins has been noted previously [23]. As mentioned above, the *exsF *and *exsG *genes are located close together (Fig. 2). This arrangement suggests that ExsF is a substrate of ExsG, which in turn could explain the good match between these proteins.

Besides phytochromes, *A. tumefaciens *contains another putative photoreceptor, a flavoprotein that belongs of the cryptochrome/photolyase group. Cryptochromes and photolyases are homologous proteins that serve as photoreceptors and catalyze light-dependent DNA repair mechanisms, respectively. In *A. tumefaciens*, the protein annotated as DNA photolyase (gi: 17935123) has also been classified as cryptochrome [31], but functional details are as yet unclear. In plants, the signal transduction pathways of cryptochromes and phytochromes are intertwined [32]. For the plant *Arabidopsis thaliana *it has been reported that a cryptochrome interacts directly with a phytochrome [33]. For these reasons, the co-distribution of phytochromes and cryptochromes/photolyases was of particular interest. In *A. tumefaciens*, there is a good match between Agp1 as query and the DNA photolyase (cryptochrome) as target; the latter is placed at positions 156 and 17 in the co-distribution tables (see Additional files 1 and 2). There is no significant co-distribution between Agp2 and cryptochrome/photolyase (see Additional files 3 and 4). In *Synechocystis *and *P. aeruginosa*, the co-distribution between phytochromes and cryptochromes/photolyases is rather poor (see Additional files 13, 14, 15 and 16), and in *D. radiodurans*, cryptochromes/photolyases seem to be absent (see Additional files 11 and 12).

The Agp1 lists were inspected for further candidates that might share common functions with this phytochrome. One striking observation was that either two or three glutamate synthase large subunits are among the first 10 proteins in the co-distribution tables (see Additional files 1 and 2). There are three large and one small subunits of this enzyme in *A. tumefaciens*. With Agp2 as query, none of the glutamate synthase subunits appeared among the first proteins in the co-distribution table (see Additional file 2). However, in the case of *D. radiodurans, Synechocystis *and *P. aeruginosa *phytochromes, cross-correlation with glutamate synthase subunits is also obvious (see Additional files 11, 12, 13, 14, 15, 16). In both *Synechocystis *Cph1 tables, two ferredoxin-dependent glutamate synthases are found among the top 60; in the *D. radiodurans *tables, the large subunit of glutamate synthase is placed among the top 50; and in the *P. aeruginosa *tables, the large subunit is found at positions 74 and 123.

Another enzyme of amino acid metabolism, methionine synthase, is also located at the top of the Agp1 lists, namely at positions 3 and 87 (see Additional files 1 and 2). Again, there is no significant co-distribution between Agp2 and this protein (see Additional files 3 and 4), but with the other phytochromes (see Additional files 11 to 16) the co-distribution is in the range of glutamate synthase (Table 5).

## Discussion

For a given target protein, the co-distribution table shows which proteins have similar or equal BLAST-homolog distributions among a set of species. These tables can be used to test for the co-distribution of protein couples with known functions or to find protein partners with a yet unknown mutuality, given that the relationship has led to co-evolution of these proteins. When the photosynthetic protein D1 was chosen as query protein, many other photosynthesis-related proteins appeared among the first ca. 150 proteins of the co-distribution tables. The ratio between these proteins and those that are not related to photosynthesis is > 3:1. This result implies that the chances of finding protein couples with related functions by the method presented here are rather high.

Two classes of proteins are known to act together with bacterial phytochromes: response regulators, which are trans-phosphorylated by the phytochrome histidine kinase subunit, and heme oxygenases, the enzymes that catalyse the last or the second last step in phytochrome chromophore biosynthesis. Depending on the species, genes for either protein may be found next to the phytochrome genes [34]. Heme oxygenases appear in rather low positions in all the phytochrome co-distribution tables. A co-evolutionary relationship between these proteins is thus not supported by the present study. Phylogenetic analyses imply that cyanobacterial heme oxygenases are of different origin from proteobacterial homologs, whereas bacterial phytochromes seem to share one common origin [35,36]. This might explain the rather large distance between the two proteins in the co-distribution analysis. The co-distribution between Agp1 (Table 3)/Cph1 (Table 4) and their cognate response regulators is in general rather good. In the phytochrome co-distribution tables, other response regulators are found higher in the list. An unambiguous identification of the cognate response regulator by the present approach is thus not expected. However, this approach could reduce the number of proteins to be analysed for those species where the response regulator is yet to be identified. In *P. aeruginosa*, the cognate phytochrome response regulator cannot be deduced from the gene arrangement. According to the list of *P. aeruginosa *proteins, there are 56 response regulators in this species; an initial biochemical screen could focus on those placed at the tops of the co-distribution lists.

The rather high frequency of histidine kinases and response regulators among those proteins listed at the tops of the phytochrome co-distribution tables suggests that bacterial phytochromes and other histidine kinases act together in a complex intracellular network. The common model of two-component signalling predicts that histidine kinases act as homodimers and that they specifically transfer phosphate to one cognate response regulator [37]. However, more complex interactions might exist in the natural host. (i) Two different histidine kinase monomers could form heterodimers. For the plant *A. thaliana *it has been shown that four of the five phytochromes can form heterodimers, most likely by their histidine-kinase-like subunits [38]. (ii) Different response regulators might function as substrates of the histidine kinase. (iii) The downstream signalling pathways of different input histidine kinases could merge. For signal transduction in which bacterial phytochromes are involved, none of these possibilities has yet been tested.

In addition to proteins with regulatory functions, the enzyme glutamate synthase is of particular interest. In *A. tumefaciens*, there are three large and one small glutamate synthase subunits. Depending on the E-value of the global BLAST analysis, either two or three of these proteins were among the top 10 in the co-distribution tables. With the exception of Agp2, all other phytochromes in the present study have co-distributed glutamate synthases. At least one glutamate synthase (subunit) is placed among the top 100 in the co-distribution lists. In the cyanobacterium *Synechocystis*, the ferredoxin-dependent enzyme matches better with the phytochrome Cph1 query protein than the NADH-dependent enzyme. In plants, where phytochrome action has been analysed over decades, there are also ferredoxin-dependent and NADH-dependent glutamate synthases. The ferredoxin-dependent enzyme is located in the plastid, where it acts together with glutamine synthase to incorporate ammonium (NH_4_^+^) into glutamine and glutamate. Ammonium is formed from nitrite (NO_2_^-^) by nitrite reductase, which like glutamate synthase is directly coupled to the photosynthetic electron cascade in the chloroplast via ferredoxin. The expression of all three enzymes and the cytosolic nitrate reductase, which catalyses the conversion of nitrate into nitrite, is light-regulated by phytochrome [39-43].

In cyanobacteria, enzymes of ammonium assimilation seem to be regulated by ammonium, but not by light [44]. Light control of gene transcription was analysed by RNA profiling in wild type and Cph1 and Cph2 mutants of *Synechocystis *[15]. In these studies, no influence of Cph1 on the abundance of glutamate synthase mRNA was found (T. Börner and T. Hübschmann, personal communication). However, the expression of GifA, a regulatory protein of glutamine synthase, which acts in cooperation with glutamate synthase in the "GOGAT cycle", seems be under the control of Cph1 and Cph2, as deduced from expression profiling results on the double mutant [15]. It could therefore be that the GOGAT cycle is indirectly under the control of phytochromes in *Synechocystis*.

The present data imply that bacterial phytochromes might contribute to the regulation of glutamate synthase in other prokaryotic species as well. The fact that phytochrome homologs and glutamate synthase homologs are found in similar sub-sets of species points to a common and ancient link between these two groups of proteins.

How can these proteins be connected? Glutamate is a key molecule of nitrogen metabolism. Glutamate and glutamine are the first amino acids in which ammonium is fixed into organic matter. Glutamate serves as nitrogen source and in most species also as a carbon source for porphyrins. In the "tRNA pathway", realized in the majority of bacteria and plants, glutamate-tRNA is used as substrate for the synthesis of δ aminolevulinic acid, which is the key molecule in porphyrin synthesis. In α proteobacteria (including *A. tumefaciens*), yeast and mammals, δ aminolevulinic acid is formed from glycine and succinate [45]. In this case, their porphyrins obtain only the amino group of glutamate, which is transferred to serine, the substrate of glycine synthesis [46]. Owing to their extended π-electron systems, porphyrins absorb visible light, predominantly in the longer wavelength regions. The biological functions of many porphyrins, for example chlorophylls in photosynthetic organisms, are directly related to light absorption; other porphyrins such as heme are involved in electron transport or redox reactions. Light absorbed by free porphyrins, leading to photosensitization, can have damaging effects by generating reactive oxygen species. Thus, the synthesis of porphyrins by light-exposed cells must be tightly regulated. Not only photosynthetic organisms but also other organisms that are exposed to sunlight might benefit from light regulation of porphyrin synthesis. The histidine kinase activity of bacterial phytochromes depends on light and the presence of the bilin chromophore. Therefore, phytochromes may also be regarded as sensors for the end product of porphyrin biosynthesis. It therefore seems plausible that phytochromes might have evolved as regulators of porphyrin synthesis.

A possible connection between phytochrome and methionine synthase is less evident. A literature survey gave no indication of phytochrome-mediated regulation of methionine synthase expression. If glutamate synthase catalyses an early step in the amino acid metabolism network, methionine synthase catalyses a late step [46,47]. Methionine, like glutamate, serves as substrate for other enzymatic reactions besides protein synthesis: the activated form, S-adenosylmethionine, is used for methylation reactions including DNA [48] and protein methylation, and for the synthesis of the gaseous hormone ethylene in land plants [49]. Methionine synthase is also important for regenerating methionine from S-adenosyl-homocysteine, the breakdown product of the methylation reaction. It could be that DNA methylation protects DNA from UV damage [50] and that the turnover of methionine is therefore higher in the light than in the dark. In this way, phytochrome could have come into play.

Although such scenarios on co-evolution are speculative, the present co-distribution data might help to gain a better understanding of phytochrome function in bacteria. The co-distribution lists contain other proteins besides those discussed in this article that might share common functions with phytochromes. In combination with genome, proteome and mutant studies, the method presented here can give clues to the evolution of signal transduction, metabolism and other cellular functions. In the present approach, only one digit was used to express protein homology (homologous or not homologous). This decision was based on the E-values of the BLAST analyses. Co-distribution analyses based on graded information about protein homology might give even more precise results. In addition, information about the length and position of homologous sequences could be included. BLAST is a heuristic algorithm, designed for fast database searches. If the number of protein sequences is not too great, accurate methods for sequence comparison can also be chosen [51]. The time required for the global BLAST analyses was approximately 2 weeks on a standard desktop computer with 2 GByte RAM. With faster machines, is seems realistic to test each possible combination of two protein sequences in a database containing 400000 sequences by non-heuristic alignment algorithms.

## Conclusion

The co-distribution analysis has allowed a deeper insight to be gained into possible evolutionary relationships. Controls with D1 as query protein show that the method identifies other proteins with related functions. The present studies have allowed the possible relationships between bacterial phytochromes and other specific proteins, such as response regulators and histidine kinases, to be tested. With glutamate synthase, a protein was identified that might be evolutionarily linked to *Agrobacterium *phytochrome Agp1 and phytochromes of other species. The method presented thus helps to guide the design of molecular studies.

## Methods

Annotated 389373 protein sequences from 138 bacteria with sequenced genomes were downloaded from the FASTA databases given under the NCBI web site [52]. A list of all species and their NCBI tax-id is given in Additional file 21. The header of each protein sequence has several fields in which the gi number, the protein function and the species name are given. Using the Linux "sed" program, these headers were modified so that the species TaxID [53] was included in each header. These modified FASTA files were concatenated and converted in one BLAST database using the formatdb program (see [54] for documentation of BLAST). Then two global BLAST analyses were performed on the local computer; the protein sequences from each modified FASTA file were used as inputs. In the first global BLAST analysis, the E-value was set to 0.000001; in the second analysis the default E-value 10 was used. The tabulated output data of the global analyses (using the BLAST-m 8 parameter) were stored in files that were named according to the input files. In this way, the results of the global BLAST analyses could be used for species-wide comparisons. In the next step, superfluous information was deleted using a PERL script. The stripped files contain the gi number and TaxID of the query proteins, and for each query protein a list of hits with the gi number, TaxID and bitscore value of each hit. The bitscore information was not used during subsequent analyses but might help for later refinements.

For co-distribution analyses, the name and gi number of the query protein (most often a phytochrome) and its hit-list were stored in a separate file, which was used as input for comparison with all other proteins of the same species. For these comparisons, a PERL script was written. This script lists the gi numbers of the target proteins, the number of species in which homologs were found and the number of hit species that were identical with the query protein. Column "e" of the list indicates whether the target protein is a "direct homolog" of the query protein. Another PERL script was used that adds the annotated protein name of each protein according to its gi number.

The probability *p *of the co-distribution found was calculated by the formula

p=(lm)(n−lk−m)(nk)=(l!(l−m)!⋅m!)((n−l)!(n−l−k+m)!(k−m)!)(n!(n−k)!⋅k!)=l!⋅(n−l)!⋅(n−k)!⋅k!n!⋅(l−m)!⋅m!⋅(n−l−k+m)!(k−m)!
 MathType@MTEF@5@5@+=feaafiart1ev1aaatCvAUfKttLearuWrP9MDH5MBPbIqV92AaeXatLxBI9gBaebbnrfifHhDYfgasaacH8akY=wiFfYdH8Gipec8Eeeu0xXdbba9frFj0=OqFfea0dXdd9vqai=hGuQ8kuc9pgc9s8qqaq=dirpe0xb9q8qiLsFr0=vr0=vr0dc8meaabaqaciaacaGaaeqabaqabeGadaaakeaacqWGWbaCcqGH9aqpdaWcaaqaamaabmaaeaqabeaacqWGSbaBaeaacqWGTbqBaaGaayjkaiaawMcaamaabmaaeaqabeaacqWGUbGBcqGHsislcqWGSbaBaeaacqWGRbWAcqGHsislcqWGTbqBaaGaayjkaiaawMcaaaqaamaabmaaeaqabeaacqWGUbGBaeaacqWGRbWAaaGaayjkaiaawMcaaaaacqGH9aqpdaWcaaqaamaabmaabaWaaSaaaeaacqWGSbaBcqGGHaqiaeaacqGGOaakcqWGSbaBcqGHsislcqWGTbqBcqGGPaqkcqGGHaqicqGHflY1cqWGTbqBcqGGHaqiaaaacaGLOaGaayzkaaWaaeWaaeaadaWcaaqaaiabcIcaOiabd6gaUjabgkHiTiabdYgaSjabcMcaPiabcgcaHaqaaiabcIcaOiabd6gaUjabgkHiTiabdYgaSjabgkHiTiabdUgaRjabgUcaRiabd2gaTjabcMcaPiabcgcaHiabcIcaOiabdUgaRjabgkHiTiabd2gaTjabcMcaPiabcgcaHaaaaiaawIcacaGLPaaaaeaadaqadaqaamaalaaabaGaemOBa4MaeiyiaecabaGaeiikaGIaemOBa4MaeyOeI0Iaem4AaSMaeiykaKIaeiyiaeIaeyyXICTaem4AaSMaeiyiaecaaaGaayjkaiaawMcaaaaacqGH9aqpdaWcaaqaaiabdYgaSjabcgcaHiabgwSixlabcIcaOiabd6gaUjabgkHiTiabdYgaSjabcMcaPiabcgcaHiabgwSixlabcIcaOiabd6gaUjabgkHiTiabdUgaRjabcMcaPiabcgcaHiabgwSixlabdUgaRjabcgcaHaqaaiabd6gaUjabcgcaHiabgwSixlabcIcaOiabdYgaSjabgkHiTiabd2gaTjabcMcaPiabcgcaHiabgwSixlabd2gaTjabcgcaHiabgwSixlabcIcaOiabd6gaUjabgkHiTiabdYgaSjabgkHiTiabdUgaRjabgUcaRiabd2gaTjabcMcaPiabcgcaHiabcIcaOiabdUgaRjabgkHiTiabd2gaTjabcMcaPiabcgcaHaaaaaa@B254@

where *k *is the number of species in which homologs of the query protein were found, *l *is the number of species with homologs of the target protein, *m *is the number of hit species with homologs to either protein, and *n *is the total number of species (= 138). If a random sample from a population of *n *members is taken *l *times without replacement, the probability that *m *samples belong to a sub-set that has *k *members is *p*.

Finally, the results were sorted according to this probability. The parameter abbreviations *k*, *l*, *m*, *n *and *p *are also used in the co-distribution tables, which are given as additional information. Since the query protein is also addressed as target protein and has the lowest *p *value, it always appears at the top of the list – unless there are other proteins that are found in exactly the same species; in this case the query protein is placed manually at the top of the list.

## Abbreviations

Agp1 and 2, phytochromes from *Agrobacterium tumefaciens*; BhpP, bacteriophytochrome proteins; Cph1, cyanobacterial phytochrome 1; AgR, response regulator of *Agrobacterium phytochrome*

## Authors' contributions

TL developed the method, loaded and modified the protein database files, performed local BLAST runs, wrote the PERL scripts, analysed the results and wrote the manuscript.

## Additional files

Co-distribution tables sorted according to the co-distribution probability *p*.

## Supplementary Material

Additional File 1 Sorted list of co-distribution results based on a low E-value global BLAST analysis, *A. tumefaciens *phytochrome Agp1 as query and all *A. tumefaciens *proteins as target. Click here for file

Additional File 2 Sorted list of co-distribution results based on a high E-value global BLAST analysis, *A. tumefaciens *phytochrome Agp1 as query and all *A. tumefaciens *proteins as target. Click here for file

Additional File 3 Sorted list of co-distribution results based on a low E-value global BLAST analysis, *A. tumefaciens *phytochrome Agp2 as query and all *A. tumefaciens *proteins as target. Click here for file

Additional File 4 Sorted list of co-distribution results based on a high E-value global BLAST analysis, *A. tumefaciens *phytochrome Agp2 as query and all *A. tumefaciens *proteins as target. Click here for file

Additional File 5 Sorted list of co-distribution results based on a low E-value global BLAST analysis, *A. tumefaciens *response regulator AgR as query and all *A. tumefaciens *proteins as target. Click here for file

Additional File 6 Sorted list of co-distribution results based on a high E-value global BLAST analysis, *A. tumefaciens *response regulator AgR as query and all *A. tumefaciens *proteins as target. Click here for file

Additional File 7 Sorted list of co-distribution results based on a low E-value global BLAST analysis, *A. tumefaciens *response regulator ExsF as query and all *A. tumefaciens *proteins as target. Click here for file

Additional File 8 Sorted list of co-distribution results based on a high E-value global BLAST analysis, *A. tumefaciens *response regulator ExsF as query and all *A. tumefaciens *proteins as target. Click here for file

Additional File 9 Sorted list of co-distribution results based on a low E-value global BLAST analysis, *A. tumefaciens *histidine kinase ExsG as query and all *A. tumefaciens *proteins as target. Click here for file

Additional File 10 Sorted list of co-distribution results based on a high E-value global BLAST analysis, *A. tumefaciens *histidine kinase ExsG as query and all *A. tumefaciens *proteins as target. Click here for file

Additional File 11 Sorted list of co-distribution results based on a low E-value global BLAST analysis, *D. radiodurans *phytochrome BphP as query and all *D. radiodurans *proteins as target. Click here for file

Additional File 12 Sorted list of co-distribution results based on a high E-value global BLAST analysis, *D. radiodurans *phytochrome BphP as query and all *D. radiodurans *proteins as target. Click here for file

Additional File 13 Sorted list of co-distribution results based on a low E-value global BLAST analysis, *P. aeruginosa *phytochrome BphP as query and all *P. aeruginosa *proteins as target. Click here for file

Additional File 14 Sorted list of co-distribution results based on a high E-value global BLAST analysis, *P. aeruginosa *phytochrome BphP as query and all *P. aeruginosa *proteins as target. Click here for file

Additional File 15 Sorted list of co-distribution results based on a low E-value global BLAST analysis, *Synechocystis *PCC 6803 phytochrome Cph1 as query and all *Synechocystis *proteins as target. Click here for file

Additional File 16 Sorted list of co-distribution results based on a high E-value global BLAST analysis, *Synechocystis *PCC 6803 phytochrome Cph1 as query and all *Synechocystis *proteins as target. Click here for file

Additional File 17 Sorted list of co-distribution results based on a low E-value global BLAST analysis, the *Synechocystis *PCC 6803 photosynthesis protein D1 as query and all *Synechocystis *proteins as target. Click here for file

Additional File 18 Sorted list of co-distribution results based on a high E-value global BLAST analysis, the *Synechocystis *PCC 6803 photosynthesis protein D1 as query and all *Synechocystis *proteins as target. Click here for file

Additional File 19 Sorted list of co-distribution results based on a low E-value global BLAST analysis, *Synechocystis *PCC 6803 response regulator Rcp1 as query and all *Synechocystis *proteins as target. Click here for file

Additional File 20 Sorted list of co-distribution results based on a high E-value global BLAST analysis, *Synechocystis *PCC 6803 response regulator Rcp1 as query and all *Synechocystis *proteins as target. Click here for file

Additional File 21 Species names of all 138 bacteria used for the co-distribution analysis. Click here for file

## Figures and Tables

**Table 1:** Co-distribution analysis result of the example outlined in Fig. 1

Query protein A1 from species A							
global BLAST run with E value of 0.000001							
gi A1 taxid A, BLAST homologs found in k = 4 species							
a: sorting range							4
b: gi of target protein							6
c: taxid of target protein							(above values for k and n for calculation of p)
l: number of species in which BLAST homologs were found							
m: number of species identical with query protein							
p: probability for co-distribution							
e: 1 if the target protein is a direct BLAST ortholog of the query protein							
f: protein annotation as							
a	b	c	l	m	p	e	f

1	A1	A	4	4	6.67E-02	1	function one protein
2	A6	A	3	3	2.00E-01	0	function six protein
3	A8	A	2	2	4.00E-01	0	function eight protein
2	A2	A	4	3	5.33E-01	1	function two protein
5	A4	A	4	3	5.33E-01	0	function four protein
6	A5	A	2	1	5.33E-01	0	function five protein
7	A9	A	2	1	5.33E-01	0	function nine protein
8	A3	A	3	2	6.00E-01	0	function three protein
9	A7	A	3	2	6.00E-01	0	function seven protein
10	A0	A	1	1	6.67E-01	0	function zero protein

**Table 2:** Summary of all co-distribution analyses. Abbreviations for species names: S6803, *Synechocystis *PCC 6803; Agrtu, *Agrobacterium tumefaciens*; Deira, *Deinococcus radiodurans*; Pseae, *Pseudomonas aeruginosa*. In cases where the results are given by two numbers separated by "/", the first number stands for the number of proteins that are listed among the first 100 of the co-distribution tables, while the second number stands for the total of all proteins in this organisms. The numbers in parentheses are the corresponding % values.

Query protein; species		global BLAST with E value of 0.000001	global BLAST with E value of 10
D1 (photosynthesis); S6803	number of proteins in analysis	3153	3166
	BLAST homologs in number of species	6	16
	Remarks	142 proteins have the same distribution as D1, for details see text	see text
Agp1 (phytochrome 1); Agrtu	number of proteins in analysis	5091	5096
	BLAST homologs in number of species	65	65
	co-distributed "two component sensors"	20/55 (20%/1.08%)	18/55 (18%/1.08%)
	co-distributed "two component response regulators"	8/48 (8%/0.94%)	7/48 (7%/0.94%)
	co-distributed "transcriptional regulators"	13/312 (13%/6.12%)	32/312 (32%/6.12%)
Agp2 (phytochrome 2); Agrtu	number of proteins in analysis	5091	5096
	BLAST homologs in number of species	15	61
	co-distributed "two component sensors"	9/55 (9%/1.08%)	15/55 (15%/1.08%)
	co-distributed "two component response regulators"	1/48 (1%/0.94%)	12/48 (12%/0.94%)
	co-distributed "transcriptional regulators"	4/312(4%/6.12%)	15/312(15%/6.12%)
AgR (response regulator); Agrtu	number of proteins in analysis	5091	5096
	BLAST homologs in number of species	19	72
	co-distributed "two component sensors"	20/55 (20/1.08)	24/55 (24/1.08)
	co-distributed "two component response regulators"	4/48 (4%/0.94%)	5/48 (7%/0.94%)
	co-distributed "transcriptional regulators"	1/312 (1%/6.12%)	10/312 (10%/6.12%)
ExsF (response regulator); Agrtu	number of proteins in analysis	5091	5096
	BLAST homologs in number of species	7	10
	co-distributed "two component sensors"	6/55 (6/1.08)	13/55 (13/1.08)
	co-distributed "two component response regulators"	2/48 (2%/0.94%)	9/48 (13%/0.94%)
	co-distributed "transcriptional regulators"	5/312 (5%/6.12%)	37/312 (37%/6.12%)
Query protein; species		global BLAST with E value of 0.000001	global BLAST with E value of 10
ExsG (histidine kinase); Agrtu	number of proteins in analysis	5091	5096
	BLAST homologs in number of species	17	80
	co-distributed "two component sensors"	10/55 (10/1.08)	20/55 (20/1.08)
	co-distributed "two component response regulators"	3/48 (3%/0.94%)	6/48 (6%/0.94%)
	co-distributed "transcriptional regulators"	5/312 (5%/6.12%)	22/312 (22%/6.12%)
BhpP (phytochrome); Deira	number of proteins in analysis	3176	3182
	BLAST homologs in number of species	30	57
	co-distributed "histidine kinases"	13/19 (13%/0.60%)	12/19 (13%/0.60%)
	co-distributed "response regulators"	11/26(11%/0.82%)	6/26(6%/0.82%)
BhpP (phytochrome); Pseae	number of proteins in analysis	5549	5566
	BLAST homologs in number of species	63	69
	co-distributed "two-component sensors"	20/51 (20%/0.92%)	24/51 (24%/0.92%)
	co-distributed "two-component response regulators"	19/56 (19%/1.01%)	16/56 (16%/1.01%)
Cph1 (phytochrome); S6803	number of proteins in analysis	3153	3166
	BLAST homologs in number of species	62	62
	co-distributed "histidine kinase"	15/28 (15%/0.80%)	13/28 (13%/0.80%)
	co-distributed "AraC/PatA/CheY/NarL/OmpR subfamily"	16/31 (16%/0.89%)	17/31 (17%/0.89%)
Rcp1 (response regulator); S6803	number of proteins in analysis	3153	3166
	BLAST homologs in number of species	21	62
	co-distributed "histidine kinase"	8/27 (8%/0.77%)	17/27 (17%/0.77%)
	co-distributed "AraC/PatA/CheY/NarL/OmpR subfamily"	1/31 (1%/0.89%)	22/31 (22%/0.89%)

**Table 3:** Co-distribution of selected *A. tumefaciens* proteins. The first column lists the names of the query proteins, and the first line lists the names of the target proteins. Numbers indicate the position of the target protein in the co-distribution lists. The first and second numbers are from global BLAST analysis with E values of 0.000001 and 10, respectively. Numbers < 100 are printed in bold, if both numbers are < 100, they are underlined.

query\target	Agp1	Agp2	AgR	ExsF	ExsG
Agp1	1/1	2237/232	321/**30**	2823/1336	2309/577
Agp2	2143/**35**	1/1	348/184	**41/2**	**4/6**
AgR	**5/52**	**34**/644	1/1	190/420	**26**/599
ExsF	2726/1513	**27/5**	1083/367	1/1	**68/15**
ExsG	1687/253	**5/7**	180/260	**61/4**	1/1

**Table 4:** Co-distribution of Cph1 and Rcp1 from *Synechocystis* PCC6803. Co-distribution of Cph1 and Rcp1, presented as in Table 3.

query\target	Cph1	Rcp1
Cph1	1/1	220/**59**
Rcp1	**4**/102	1/1

**Table 5:** Phytochromes as query and selected target proteins. The co-distribution of selected proteins, presented as in Table 3.

query\target	photolyase/cryptochrome	glutamate synthases	methionine synthase
AgrtuAgp1	156/17	4,6,2552 (923)/3,5,10 (988)^1^	3/87
AgrtuAgp2	1855/2054	2767,2841,4656 (2999)/144,201,247 (950)^1^	1749/373
DeiraBphP	n.a.	45 (614)/13 (1593) ^2^	19/153^4^
PseaBphP	655/958	74 (273)/123 (235) ^2^	19/419
S6803Cph1	420,421/630,756	7,8 (311)/12,53 (916) ^3^	5/30^4^

^1 ^three large subunits and one small subunit (positions of the latter are given in brackets)

^2 ^one large subunit and one small subunit (positions of the latter are given in brackets)

^3 ^two ferredoxin-dependent and one NADH-dependent enzymes (positions of the latter are given in brackets)

^4 ^designated as 5-methyltetrahydrofolate-homocysteine methyltransferase
