# Supplementary material for: A computational approach to discovering the functions of bacterial phytochromes by analysis of homolog distributions
Source: BMC Bioinformatics. 2006 Mar 16;7:141. doi: 10.1186/1471-2105-7-141 (PMC1552090; doi:10.1186/1471-2105-7-141)
Supplement: Additional File 21 — Species names of all 138 bacteria used for the co-distribution analysis. [file 1471-2105-7-141-S21.pdf]

Additional file 21- List of used bacteria

Species names of all 138 bacteria used for the co-distribution analysis. TaxIDs of each species are given in brackets. The six cyanobacterial species are given in bold letters. Those species in which Agp1 homologs were found are underlined.

Acinetobacter sp. ADP1 (62977); Aeropyrum pernix K1(272557); Agrobacterium tumefaciens str. C58 (176299); Aquifex aeolicus VF5 (224324); Archaeoglobus fulgidus DSM 4304(224325); Bacillus anthracis str. A2012 (191218); Bacillus cereus ATCC 10987 (222523); Bacillus halodurans C-125 (272558); Bacillus subtilis subsp. subtilis str. 168 (224308); Bacillus thuringiensis serovar konkukian str. 97-27 (281309); Bacteroides thetaiotaomicron VPI-5482 (226186); Bartonella henselae str. Houston-1 (283166); Bartonella quintana str. Toulouse (283165); Bdellovibrio bacteriovorus HD100(264462); Bifidobacterium longum NCC2705 (206672); Bordetella bronchiseptica RB50 (257310); Bordetella parapertussis 12822 (257311); Bordetella pertussis Tohama I (257313); Borrelia burgdorferi B31 (224326); Buchnera aphidicola str. APS (Acyrthosiphon pisum) (107806); Buchnera aphidicola str. Bp (Baizongia pistaciae) (224915); Buchnera aphidicola str. Sg (Schizaphis graminum) (198804); Campylobacter jejuni subsp. jejuni NCTC 11168 (192222); Candidatus Blochmannia floridanus (203907); Caulobacter crescentus CB15 (190650); Chlamydia muridarum (83560); Chlamydia trachomatis D/UW-3/CX (272561); Chlamydophila caviae GPIC (227941); Chlamydophila pneumoniae AR39 (115711); Chlamydophila pneumoniae CWL029 (115713); Chlorobium tepidum TLS (194439); Chromobacterium violaceum ATCC 12472 (243365); Clostridium acetobutylicum ATCC 824 (272562); Clostridium perfringens str. 13 (195102); Clostridium tetani E88 (212717); Corynebacterium diphtheriae NCTC 13129 (257309); Corynebacterium glutamicum ATCC 13032 (196627); Coxiella burnetii RSA 493 (227377); Deinococcus radiodurans R1 (243230); Desulfotalea psychrophila LSv54 (177439); Desulfovibrio vulgaris subsp. vulgaris str. Hildenborough (882); Enterococcus faecalis V583 (226185); Erwinia carotovora subsp. atroseptica SCRI1043 (218491); Escherichia coli CFT073 (199310); Escherichia coli K12 (83333); Fusobacterium nucleatum subsp. nucleatum ATCC 25586 (190304); Geobacter sulfurreducens PCA (243231); **Gloeobacter violaceus PCC 7421 (251221)**; Haemophilus ducreyi 35000HP (233412); Haemophilus influenzae Rd KW20 (71421); Halobacterium sp. NRC-1(64091); Helicobacter hepaticus ATCC 51449 (235279); Helicobacter pylori 26695(85962); Lactobacillus johnsonii NCC 533 (257314); Lactobacillus plantarum WCFS1 (220668); Lactococcus lactis subsp. lactis Il1403 (272623); Leifsonia xyli subsp. xyli str. CTCB07 (281090); Leptospira interrogans serovar Lai str. 56601 (189518); Listeria innocua Clip11262 (272626); Listeria monocytogenes EGD-e (169963); Mesorhizobium loti MAFF303099 (266835); Methanocaldococcus jannaschii DSM 2661 (243232); Methanococcus maripaludis S2 (267377); Methanopyrus kandleri AV19 (190192); Methanosarcina acetivorans C2A (188937); Methanosarcina mazei Goe1 (192952); Methanothermobacter thermautotrophicus str. Delta H (187420); Mycobacterium avium subsp. paratuberculosis str. k10 (262316); Mycobacterium bovis AF2122/97 (233413); Mycobacterium leprae TN (272631); Mycobacterium tuberculosis CDC1551 (83331); Mycoplasma gallisepticum R (233150); Mycoplasma genitalium G-37 (243273); Mycoplasma mobile 163K (267748); Mycoplasma mycoides subsp. mycoides SC str. PG1 (272632); Mycoplasma penetrans HF-2 (272633); Mycoplasma pneumoniae M129 (272634); Mycoplasma pulmonis UAB CTIP (272635); Nanoarchaeum equitans Kin4-M(228908); Neisseria meningitidis MC58 (122586); Nitrosomonas europaea ATCC 19718 (228410); **Nostoc sp. PCC 7120 (103690)**; Oceanobacillus iheyensis HTE831 (221109); Onion yellows phytoplasma OY-M (262768); Parachlamydia sp. UWE25 (264201); Photorhabdus luminescens subsp. laumondii TTO1 (243265); Pirellula sp. 1 (243090); Porphyromonas gingivalis W83 (242619); **Prochlorococcus marinus str. MIT 9313 (74547)**; Propionibacterium acnes KPA171202 (267747); Pseudomonas aeruginosa PAO1 (208964); Pseudomonas putida KT2440 (160488); Pseudomonas syringae pv. tomato str. DC300+0 (223283); Pyrobaculum aerophilum str. IM2(178306); Pyrococcus abyssi GE5(272844); Pyrococcus furiosus DSM 3638(186497); Pyrococcus horikoshii OT3(70601); Ralstonia solanacearum GMI1000 (267608); Rhodopseudomonas palustris CGA009 (258594); Rickettsia conorii str. Malish 7 (272944); Rickettsia prowazekii str. Madrid E (272947); Rickettsia typhi str. Wilmington (257363); Salmonella enterica subsp. enterica serovar Typhi Ty2 (209261); Shewanella oneidensis MR-1 (211586); Shigella flexneri 2a str. 2457T (198215); Sinorhizobium meliloti 1021 (266834); Staphylococcus aureus subsp. aureus MW2 (196620); Streptococcus agalactiae NEM316 (211110); Streptococcus mutans UA159 (210007); Streptococcus pneumoniae R6 (171101); Streptococcus pyogenes M1 GAS (160490); Streptomyces avermitilis MA-4680 (227882); Streptomyces coelicolor A3(2) (100226); Sulfolobus solfataricus P2(273057); Sulfolobus tokodaii str. 7(273063); **Synechococcus sp. WH 8102 (84588)**; **Synechocystis sp. PCC 6803 (1148)**; Thermoanaerobacter tengcongensis (119072); Thermoplasma acidophilum DSM 1728(273075); Thermoplasma volcanium GSS1(273116); **Thermosynechococcus elongatus BP-1 (197221)**; Thermotoga maritima MSB8 (243274); Thermus thermophilus HB27 (262724); Treponema denticola ATCC 35405 (243275); Treponema pallidum subsp. pallidum str. Nichols (243276); Tropheryma whipplei str. Twist (203267); Ureaplasma parvum serovar 3 str. ATCC 700970 (273119); Vibrio cholerae O1 biovar eltor str. N16961 (243277); Vibrio

parahaemolyticus RIMD 2210633 (223926); Vibrio vulnificus CMCP6 (216895); Wigglesworthia glossinidia endosymbiont of Glossina brevipalpis (36870); Wolbachia endosymbiont of Drosophila melanogaster (163164); Wolinella succinogenes DSM 1740 (273121); Xanthomonas axonopodis pv. citri str. 306 (190486); Xanthomonas campestris pv. campestris str. ATCC 33913(190485); Xylella fastidiosa 9a5c (160492); Xylella fastidiosa Temecula1 (183190); Yersinia pestis KIM (187410)
